# Intramolecular H-bonding interaction in angular 3-π-EWG substituted imidazo[1,2-a]pyridines contributes to conformational preference

**DOI:** 10.1186/1752-153X-7-20

**Published:** 2013-01-31

**Authors:** Manuel Velázquez-Ponce, Héctor Salgado-Zamora, Hugo A Jiménez-Vázquez, Maria Elena Campos-Aldrete, Rogelio Jiménez, Humberto Cervantes, Taibi Ben Hadda

**Affiliations:** 1 Departamento de Formación Básica Disciplinaria, Unidad Profesional Interdisciplinaria de Ingeniería Campus Guanajuato, Instituto Politécnico Nacional, Silao de la Victoria-Guanajuato, 36275, Mexico; 2 Departamento de Química Orgánica, Escuela Nacional de Ciencias Biológicas, Instituto Politécnico Nacional, Mexico, DF, 11340, Mexico; 3 Área de Química, Universidad Autónoma Metropolitana-Unidad Azcapotzalco, Mexico, DF, 02200, México; 4 Université Mohammed Premier, Faculté des Sciences, Laboratoire Chimie Matériaux, Oujda, 60000, Morocco

**Keywords:** Imidazo[1,2-a]pyridine, Peri effect, Hydrogen bonding, Conformational preference

## Abstract

**Background:**

The proton at position 5 of imidazo[1,2-a]pyridines substituted with an angular electron withdrawing group (EWG) at position 3, shows an unusual downfield chemical shift, which is usually explained in terms of a peri effect. However usage of this term is sometimes confusing. In this investigation, it is proposed that the aforementioned shift is in fact a combination of several factors: Anisotropy, long-distance mesomerism and an attractive intramolecular interaction of the electrostatic hydrogen bond type.

**Results:**

Theoretical calculations were performed aimed to obtain evidence of the existence of an intramolecular non-bonding interaction between H-5 and the oxygen atom of the EWG. Results derived from conformational and vibrational analysis at the DFT B3LYP/6-311++G(d,p) level of theory, the determination of Bond Critical Points derived from AIM theory, and the measurement of some geometrical parameters, support the hypothesis that the higher stability of the prevailing conformation in these molecules (that in which the oxygen of the EWG is oriented towards H-5) has its origin in an intramolecular interaction.

**Conclusion:**

Computational calculations predicted correctly the conformational preferences in angular 3-π-EWG-substituted imidazo[1,2-a]pyridines. The existence of an electrostatic hydrogen bond between H-5 and the oxygen atom of the π-EWG was supported by several parameters, including X-ray crystallography. The existence of such structural array evidently impacts the H-5 chemical shift.

## Background

**Figure 1:**
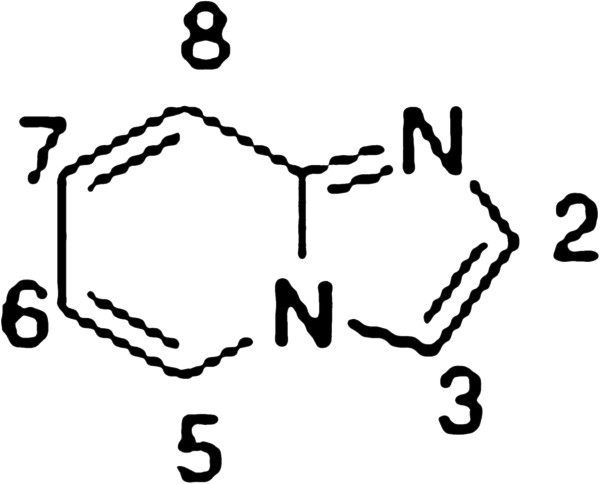
The imidazo[1,2-a]pyridine system with ring atoms numbered.

Derivatives of imidazo[1,2-a]pyridine 1 (Figure 1) are an important class of fused heterocyclic compounds. Their biological activities have been profusely investigated and as a consequence reports abound on the subject [1-3]. Theoretical calculations have also been performed on the system, aimed to explain its reactivity [4,5].

From the structural point of view, this fused heterocycle is attractive as well. Substitution at position 3 of the heterocycle with an electron-withdrawing group (EWG), for instance a nitro group, shifts H-5 to low fields (ca. δ 9.4) [6]. The H-5 of 7-methyl-3-nitroso-2-(pyridin-2-yl) imidazo[1,2-a]pyridine is shifted to δ 9.7 [7]. In the latter case, the unusual chemical shift was explained in terms of a peri effect. An X-ray analysis of this compound showed that the oxygen atom of the nitroso group is oriented towards H-5, apparently justifying an anisotropic effect as responsible for the magnetic deshielding of the peri H-5. Paudler attributed the deshielding effect on H-5 of 3-substituted imidazo[1,2-a]pyridines to a through-space electric field effect [8].

The peri effect is used to define a repulsive non-bonding interaction between substituents placed at positions 1 and 8 of the naphthalene core [9]. However, the notion of repulsion involved in the definition limits the usage of the term, which in some instances may even be confusing. Table 1 discloses the H-5 chemical shifts of several imidazo[1,2-a]pyridines substituted with various EWG at position 3.

**Table 1:** H-5 chemical shift of some imidazo[1,2-a]pyridines substituted with angular EWG at position 3

**Name**	**δ H-5**^**a **^**Exp**	**δ H-5**^**a**^	**Reference**
imidazo[1,2-a]pyridine	8.05	8.05	[8]
3-nitroso-2-phenyl imidazo[1,2-a]pyridine	9.89	9.97-10	[10]
3-formyl-2-phenyl imidazo[1,2-a]pyridine	9.68	9.66	[11]
3-formyl imidazo[1,2-a]pyridine		9.52	[12]
3-benzoyl imidazo[1,2-a]pyridine	9.75	9.75	[13]
3-acetyl-2-methyl imidazo[1,2-a]pyridine	9.58	9.81	[14]
3-(2-chloroacetyl) imidazo[1,2-a]pyridine	9.48	9.48	[13]
3-nitro imidazo[1,2-a]pyridine	9.38	9.40	[5]
3-nitro-2-(4-nitrophenyl) imidazo[1,2-a]pyridine	9.50^b^	9.95^c^	[15]

^a^ HNMR taken in CDCl_3_ unless otherwise stated. ^b^ Solvent DMSO-d6. ^c^ Solvent TFAA.

In this study an interaction between the angular EWG at position 3 of the imidazo[1,2-a]pyridine ring system and H-5 of the same molecule is demonstrated by theoretical means. It is proposed that the establishment of such interaction may promote a preferred conformation of the group at position 3, independent of the substituent at position 2. The adoption of such conformation, which definitely impacts the H-5 chemical shift, should contribute to offer a more precise explanation of the nature of the peri effect.

## Results and discussion

**Scheme 1:**
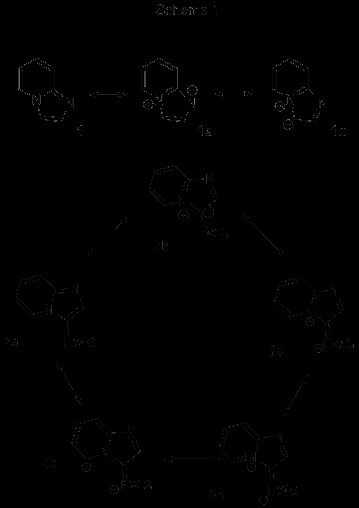
Resonance structures of imidazo[1,2-a]pyridine.

**Figure 2:**
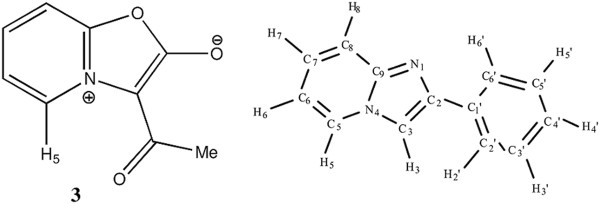
Mesoionic structure of 3-acyl oxazolo[3,2-a]pyridinium-2-olate 3 and 2-phenyl imidazo[1,2-a]pyridine with ring atoms numbered for the dihedral angle analysis.

As already mentioned, the downfield H-5 chemical shift of imidazo[1,2-a]pyridines substituted with angular EWGs at position 3, may be explained in terms of an anisotropy effect. However it is possible that the real cause might be a series of concurrent events. An analysis of mesomeric structures [16] (Scheme 1), suggests that two major contributors to the imidazo[1,2-a]pyridine resonance hybrid are those in which both, the six-membered and the five-membered rings have aromatic character (mesomeric structures 1a and 1b). The latter also suggests that position 3 is rich in electron density, a structural argument put forward to account for the high reactivity of this position towards electrophiles [17-19]. The introduction of an electron-withdrawing group (cyano, nitroso, nitro, carbonyl) at position 3, contributes to the stabilization of the π electron density by placing a charge on the electronegative atom of the electron-withdrawing group (structures 2c–d), and in doing so a long distance mesomeric effect develops a positive charge on C-5 (structure 2e). Moreover, in structures 2c–d the pyridine nitrogen is positively charged, thus creating a deshielding effect on C-5 and consequently shifting H-5 to low fields. At the same time, accumulation of electron density at the most electronegative atom of the EWG gives as a result an additional deshielding effect on H-5. For comparison, in mesoionic 3-acyl oxazolo[3,2-a]pyridinium-2-olate 3 (Figure 2) H-5 was observed at δ 9.78 as a doublet (J_5,6 =_ 6.5 Hz). The latter shift was attributed to the presence of the magnetically anisotropic acyl group at the 3 peri position [20]. The introduction of an additional electron-withdrawing group in the ring shifts H-5 slightly further down field, e.g. in 2-aryl imidazo[1,2-a]pyrimidine-3-carbaldehyde, H-5 is found at δ 9.80-9.90. [21] However, structures 2c–d suggest that an intramolecular C-H hydrogen bond is forming, but a hydrogen bond is not a repulsive but rather an attractive interaction. Thus, in the case where mesomeric or attractive interactions are participating, the term peri effect would seem inappropriate, considering the original definition that takes into account repulsive non-bonding interactions. Theoretical studies on hydrogen bonds in which the donor is a carbon atom bonded to a quaternary nitrogen atom are described in the literature [22]. In a recent study the formation of intramolecular hydrogen bonds, their effects on the structure and properties of molecules, and their impact in medicinal chemistry was analyzed [23].

**Scheme 2:**
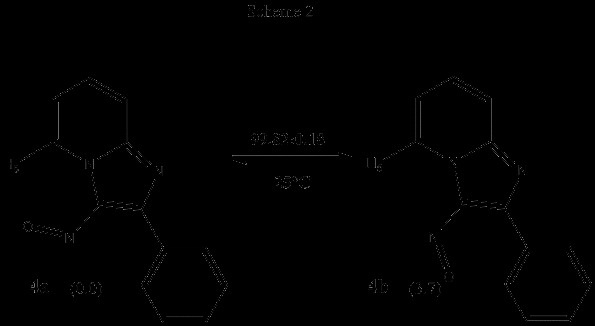
Relative energies (∆ G, kcal/mol) and conformational populations for conformers 4a and 4b.

**Figure 3:**
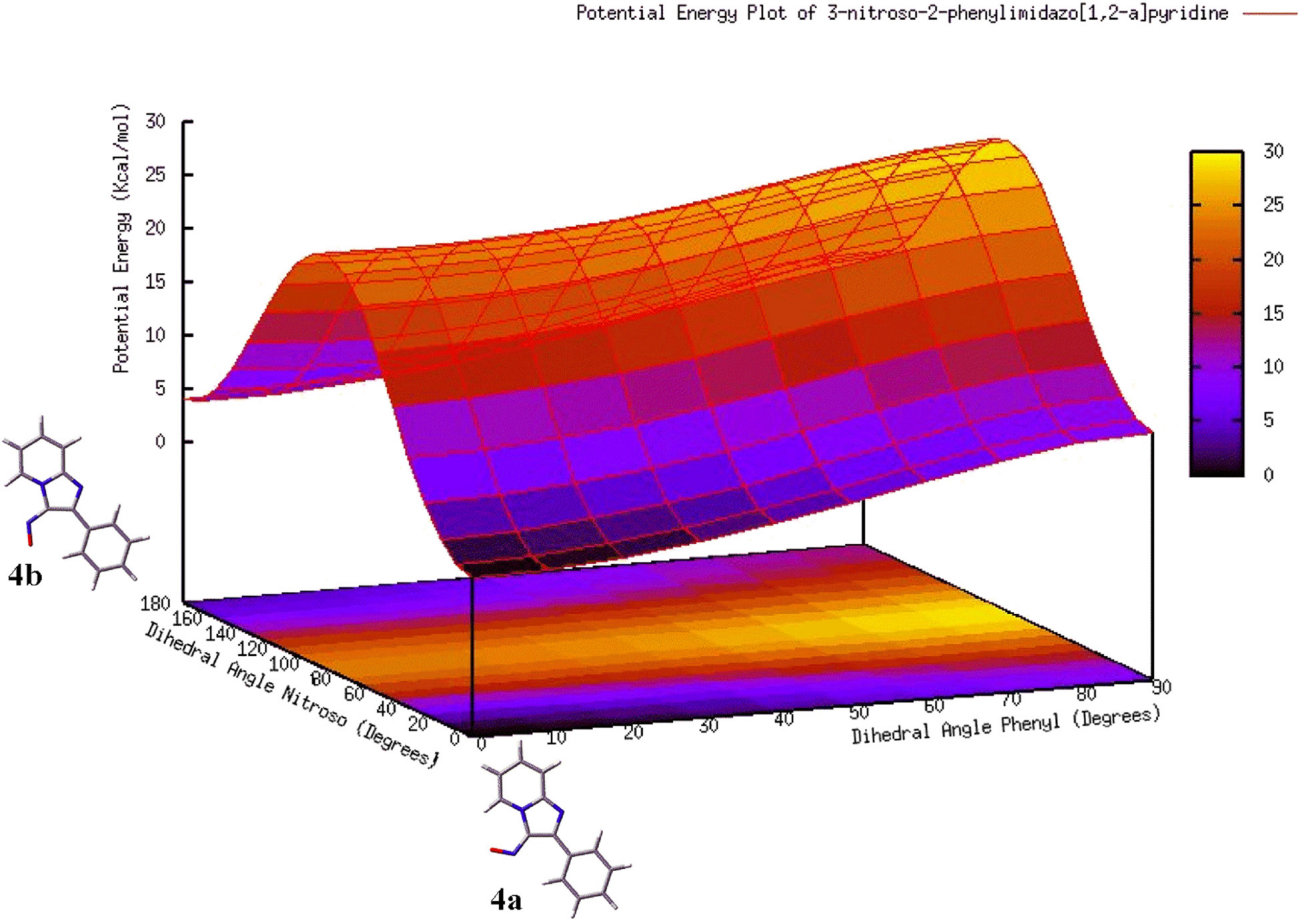
**HF/3-21G Potential energy plot of 3-nitroso-2-phenyl imidazo[1,2-a]pyridine. Potential energy (kcal/mol) vs N**_**4**_**-C**_**3**_**-N**_**nitr**_**-O**_**nitr **_**and C**_**3**_**-C**_**2**_**-C**_**1’**_**-C**_**2’**_**, dihedral angles (degrees).**

The possibility of a non-bonding H-interaction between H-5 and the oxygen atom of the angular EWG was thus analyzed. A dihedral angle screening on the C-3 substituent of 3-nitroso-2-phenyl imidazo[1,2-a]pyridine 4 and of 3-formyl-2-phenyl imidazo[1,2-a]pyridine 5 was carried out at the HF/3-21G level of theory. Structure reoptimization at the B3LYP/6-311++G(d,p) level was performed for the minimum-energy geometries found, and then these underwent vibrational analyses where no imaginary vibrational frequencies were found. In the case of 4, two minima were found, 4a,b. In these, the nitroso and phenyl groups are coplanar with the imidazo[1,2-a]pyridine ring. The global minimum was the structure in which the oxygen atom of the nitroso group is oriented towards H-5 (0.0° for the N_4_-C_3_-N_nitr_-O_nitr_ dihedral and 0.0° for the C_3_-C_2_-C_1’_-C_2’_ dihedral of the phenyl moiety). The interatomic distance between H-5 and O_NO_ was 2.197Å . This value is smaller than the sum of the van der Waals radii of the atoms (2.61 Å ). Conformer 4b, in which the nitroso oxygen points towards the phenyl system, is also coplanar (180.0° for the N_4_-C_3_-N_nitr_-O_nitr_ dihedral and 0.0° for the C_3_-C_2_-C_1’_-C_2’_ dihedral). The energy difference between these minima was determined from the electronic energies after applying thermal free energy corrections; at 298 K, ∆ G = 3.7 kcal/mol favoring 4a. From these values the conformational population of the two minima can be determined (Scheme 2). The results of the HF/3-21G dihedral angle screening already mentioned are shown as a potential energy surface in Figure 3.

**Scheme 3:**
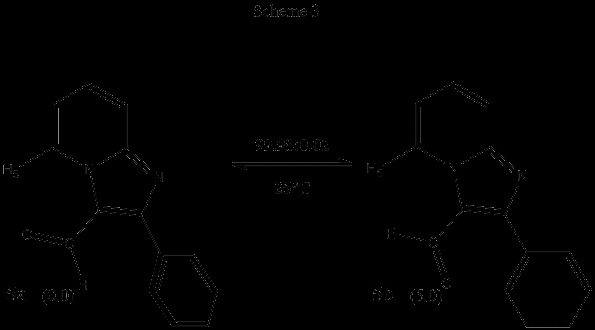
Relative energies (∆ G, kcal/mol) and conformational populations for conformers 5a and 5b.

In the case of 3-formyl-2-phenyl imidazo[1,2-a]pyridine 5, the global minimum found was conformer 5a, with the formyl oxygen atom oriented towards H-5, and slightly deviated from coplanarity relative to the heterocyclic system (6.58° for the N_4_-C_3_-C_carb_-O_carb_ dihedral). The phenyl ring is also more deviated from coplanarity with a C_3_-C_2_-C_1’_-C_2’_ dihedral angle of 36.72 degrees. The distance found between the carbonyl oxygen atom and H-5 was 2.254 Å . In conformer 5b a deviation from planarity of the formyl moiety relative to the imidazo[1,2-a]pyridine ring is present (175.91° for dihedral N_4_-C_3_-C_carb_-O_carb_) and apparently a small interaction exists between the carbonyl oxygen with a proton of the phenyl system (which is less deviated than in conformer 5a, 16.05° for dihedral C_3_-C_2_-C_1’_-C_2’_). These features imply conformational differences between the 3-nitroso and the 3-formyl-2-phenyl imidazo[1,2-a]pyridine. The distribution of conformers under equilibrium conditions at 298 K, with ΔG = 5.0 kcal/mol favoring 5a, is shown in Scheme 3.

**Figure 4:**
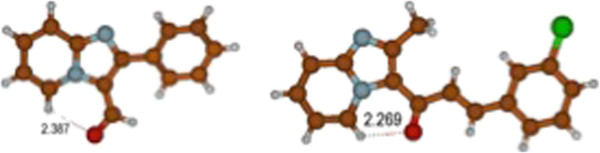
Carbonyl and peri H-5 interatomic distances (from X-ray diffraction data) in 2-phenyl imidazo[1,2-a]pyridine-3-carbaldehyde and in 3-(3-chlorophenyl)-1-(2-methyl-imidazo[1,2-a]pyridin-3-yl)prop-2-en-1-one.

As a comparison with the above results, the crystal structure of compound 5a, showed a distance of 2.39 Å between the carbonyl oxygen and H-5 and a dihedral angle between the imidazo[1,2-a]pyridine and phenyl ring of 28.61(4)° [24], which confirms the theoretical prediction of the preferred conformation. In a similar vein 3-(3-chlorophenyl)-1-(2-methyl imidazo[1,2-a]pyridin-3-yl)prop-2-en-1-one showed a distance of 2.27 Å and a dihedral angle of 7.43(1)° between the imidazopyridine ring system and the benzene ring [25], Figure 4.

**Scheme 4:**
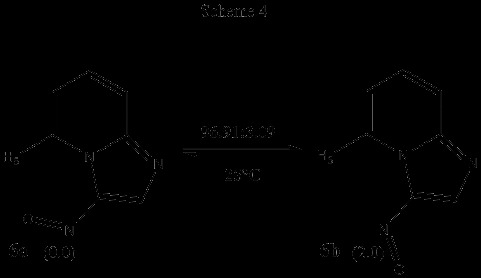
Relative energies (∆ G, kcal/mol) and conformational populations for conformers 6a and 6b.

**Figure 5:**
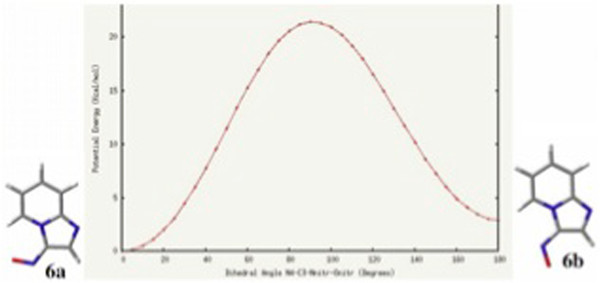
Potential energy (HF/3-21G, kcal/mol) of 3-nitroso imidazo[1,2-a]pyridine vs. dihedral angle (degrees).

In order to discard any possible influence (steric, electronic) of the adjacent phenyl ring on the adopted conformations of 4a and 5a, it was decided to carry out a similar analysis on the 2-unsubstituted 3-nitroso and 3-formyl imidazo[1,2-a]pyridines (6 and 7, respectively). Accordingly, the corresponding structures were optimized at the B3LYP/6-311++G(d,p) level of theory. In the case of 6, two minima corresponding to structural conformations in which the nitroso moiety was coplanar with the imidazo[1,2-a]pyridine nucleus were found. From these conformers, the global minimum 6a was once again, that in which the oxygen of the nitroso moiety is oriented towards H-5. An energy difference of ΔG = 2.0 kcal/mol between the minima was found, which under equilibrium conditions (298 K) should lead to the conformer population shown in Scheme 4. In Figure 5, the plot of potential energy as a function of dihedral angle is shown.

**Scheme 5:**
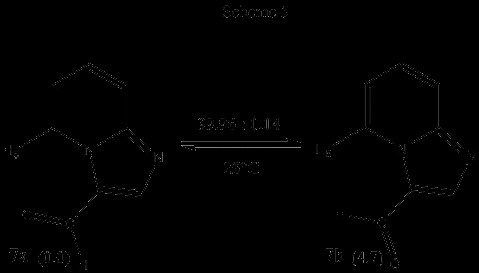
Relative energies (∆ G, kcal/mol) and conformational populations for conformers 7a and 7b.

**Figure 6:**
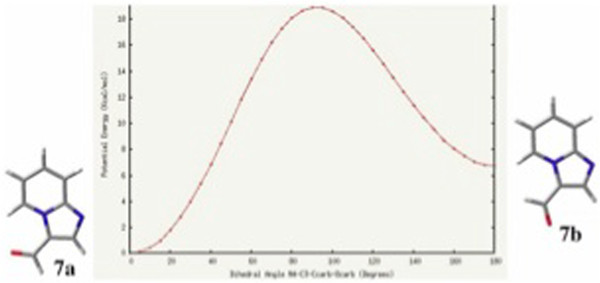
Potential energy (HF/3-21G, kcal/mol) of 3-formyl imidazo[1,2-a]pyridine vs. dihedral angle (degrees).

Similarly, two minima were found for 3-formyl imidazo[1,2-a]pyridine 7. Again, the global minimum is 7a, that in which the formyl oxygen atom is oriented towards H-5. The other minimum 7b is the conformer in which the formyl hydrogen is oriented towards H-5. An energy difference of ΔG = 4.7 kcal/mol between the minima was obtained. Thus, the distribution of conformers at equilibrium is shown in Scheme 5. Figure 6 shows the plot of potential energy vs. dihedral angle.

The interatomic distances between H-5 and O_NO_ and between H-5 and O_CHO_ were 2.265 Å and 2.321 Å , respectively. These represent smaller values than the corresponding sum of their van der Waals radii. The marked conformational preference shown by derivatives 4–7, supports the existence of an attractive intramolecular interaction between the oxygen atom of the electron-withdrawing group and H-5. Furthermore, such interaction should have a strong electrostatic character considering that the angular EWG located at the peri position would accumulate charge over the most electronegative oxygen atom. This, in turn, would lead to an attractive interaction onto H-5, which is attached to a carbon bonded to a nitrogen atom bearing a strong positive character.

### Hydrogen bonding interaction

One of the most widely accepted criteria for validating the formation of an intramolecular H···X hydrogen bond is the magnitude of the interatomic distance between the atoms involved, which should be less than the sum of their van der Waals radii [26]. However, from the theoretical point of view, and according to AIM theory [27], the requirement is the existence of a bond critical point of a particular nature [28] between the H···X atoms. Therefore in order to confirm the formation of a hydrogen interaction between H-5 and either the nitroso [C-H···O=N] or the formyl [C-H···O=C] oxygen, we carried out calculations aimed to determine the existence of bond critical points, employing the AIMAll program, starting from the wavefunctions of the described conformers obtained from the Gaussian 98 calculations.

**Figure 7:**
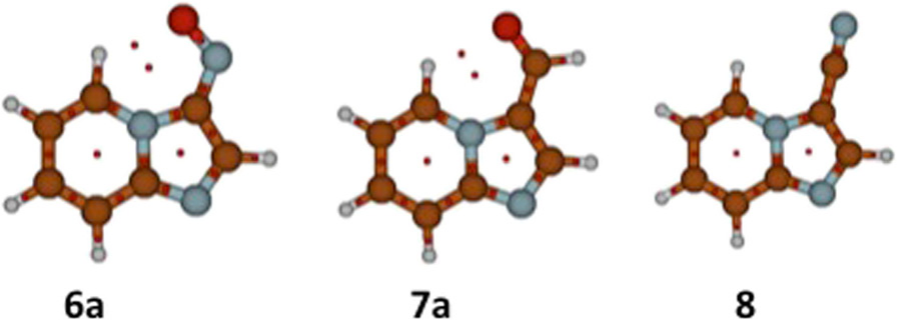
Bond Critical Points (BCPs) and Ring Critical Points (RCPs) represented as red dots for 3-nitroso imidazo[1,2-a]pyridine 6a, 3-formyl imidazo[1,2-a]pyridine 7a and 3-cyano imidazo[1,2-a]pyridine 8.

Critical points in the interatomic space were found for both global minima structures 5a and 6a, with electron densities (ρ_b_) and Laplacian of the electron density (∇^2^ρ_b_) values suggesting an electrostatic interaction [28,29] between H-5 and the oxygen atom. (Figure 7, Table 2).

**Table 2:** Topological Parameters for BCP found between H-5 and O for conformers 6a and 7a and values for an electrostatic hydrogen bond (feature values)

**Topological Parameter**	**Compound 6a**	**Compound 7a**	**Feature Value**
ρ_BCP_	0.017	0.014	0.002-0.040
∇^2^ρ_BCP_	0.060	0.049	0.024-0.139
d H· · · Y	2.26	2.32	H· · · O < 2.61

In order to have an idea of the electron withdrawing effect of the 3-angular substituent on the chemical shift of H-5, the imidazo[1,2-a]pyridine-3-carbonitrile 8, was synthesized from the corresponding imidazo[1,2-a]pyridine-3-carboxaldehyde oxime using acetic anhydride as the dehydrating agent [30]. The experimental ^1^H NMR spectrum of 8 showed that indeed H-5 was not shifted to very low fields by the introduction of the linear nitrile group, δ 8.36 (CDCl_3_). As expected, the corresponding calculations showed that no critical point was found between H-5 and the nitrile (Figure 7). This suggests that the π-EWG geometry is mandatory and that the H-5 chemical shift shown in 8 involves solely the inductive and mesomeric effects of the nitrile group.

**Figure 8:**
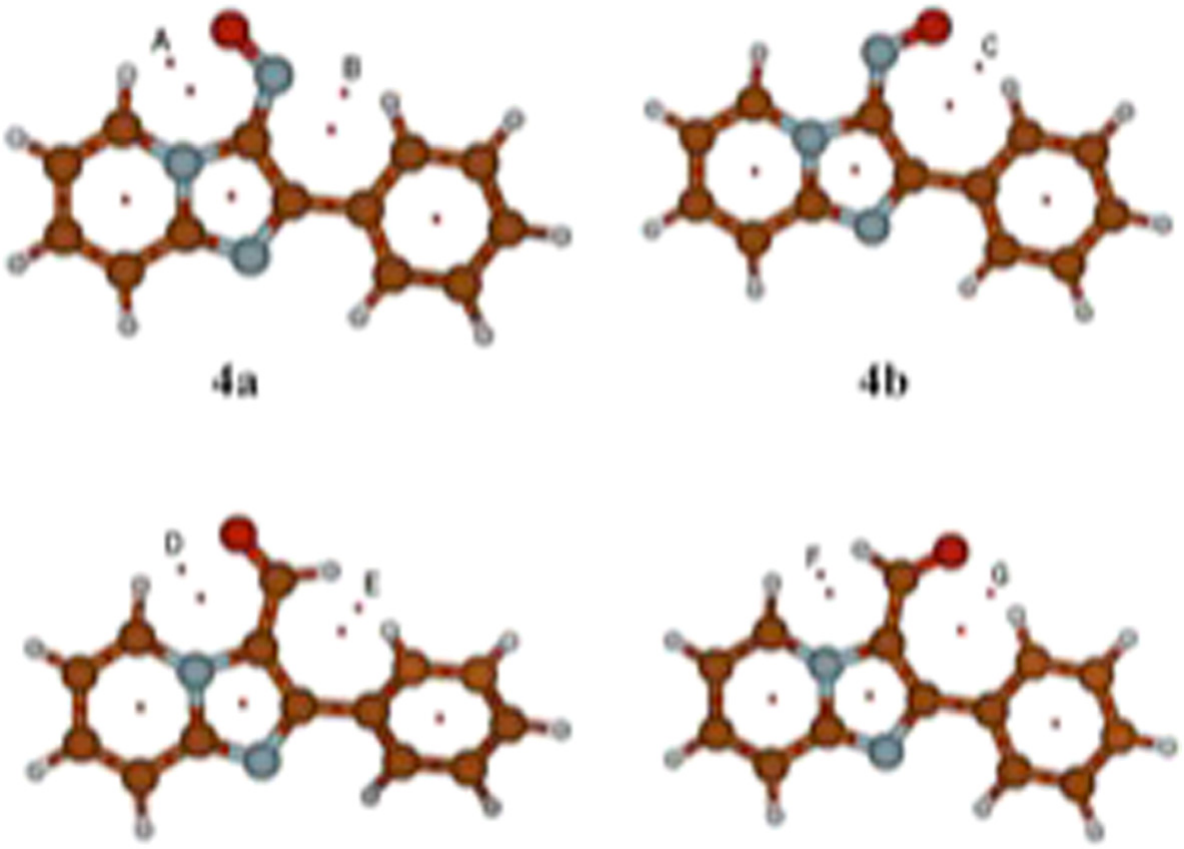
BCP’s and RCP’s for the conformers of 4 and 5.

The BCPs and RCPs determined for conformers 4a,b and 5a,b are shown in Figure 8. Note that in addition to the critical points expected for the interaction between H-5 and the most electronegative atom of the EWG (A and D for 4a, and 5a, respectively), there are other BCPs evidencing additional interactions between the group at C-3 and the rest of the molecule. In particular, the critical points C and G (4b and 5b) support a hydrogen-bond type interaction with the ortho hydrogens of the phenyl ring, as hinted before. Three additional BCPs were located: B which suggests the formation of an additional ON· · · H-Ph interaction for the nitroso group in 4a, and E and F (5b and 5b) which indicate interactions between the formyl C-H bond and the C-H bond of either C-5 or the phenyl ring. Interactions of this type have been proposed before as a new type of hydrogen bond called dihydrogen bond [31]. The topological properties of these BCPs are summarized in Table 3 along with the corresponding internuclear distances; all of them fall within the ranges expected for hydrogen bonds (see Table 2).

**Table 3:** AIM Topological Parameters for the BCPs found for the conformers of 4 and 5

**Conformer**	**BCP**	**Interaction**	**ρ**_**b**_	**∇**^**2**^**ρ**_**b**_	**d (Å)**
4a	A	C_5_-H···O=N	0.019	0.070	2.197
4a	B	Ph-H···N=O	0.015	0.050	2.337
4b	C	Ph-H···O=N	0.024	0.087	2.029
5a	D	C_5_-H···O=C	0.016	0.057	2.254
5a	E	Ph-H···H-CO	0.009	0.029	2.302
5b	F	C_5_-H···H-CO	0.012	0.043	2.027
5b	G	Ph-H···O=C	0.021	0.076	2.092

The observed chemical shifts for the peri H-5 of imidazo[1,2-a]pyridines caused by the EWG at 3, with no substituent at position 2, follow the order: PhC(O) > HC(O) > MeC(O) > O_2_N, i.e. the strongest electron-withdrawing group has a minor deshielding effect on H-5. It is possible that the observed decreased effect on the H-5 chemical shift might be a consequence of a charge distribution on the oxygen nitro atoms. The introduction of the phenyl ring shifts the H-5 signal to lower fields (Table 1).

**Figure 9:**
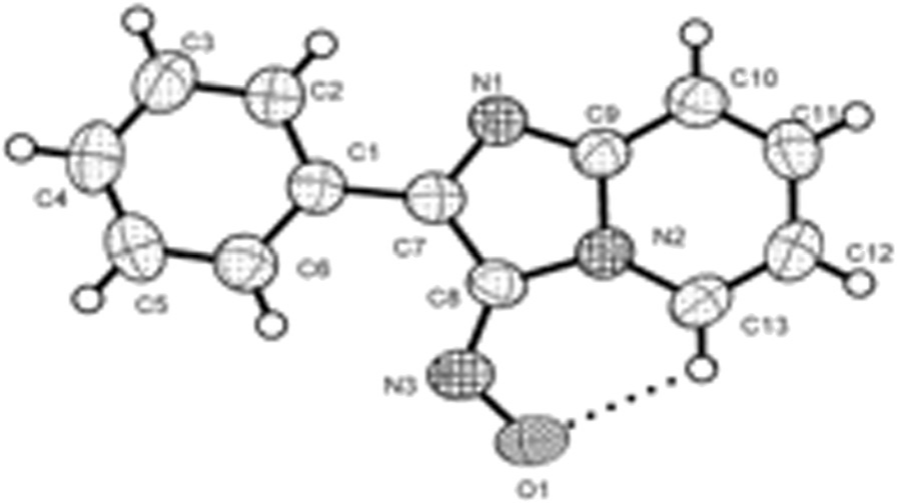
ORTEP of 3-nitroso-2-phenyl imidazo[1,2-a]pyridine 4.

Finally, an X-ray diffraction analysis performed on 3-nitroso-2-phenyl imidazo[1,2-a]pyridine 4 (Figure 9) in which the nitroso oxygen points towards H-5, further confirms the theoretical prediction of the preferred conformation in the crystalline structure (Additional file 1 and 2). It is also interesting to observe a small deviation (10.5°) of the 2-phenyl substituent from coplanarity with respect to the imidazo[1,2-a]pyridine nucleus. The theoretical calculations predicted a slight deviation from coplanarity between these two rings. It is important to emphasize that the crystalline array of 4, as well as those of other related analogues such as the ones shown in Figure 4[24,25,32], strongly supports the existence of an intramolecular hydrogen bond interaction of the C-H···O type between H-5 and the most electronegative atom of the angular electron-withdrawing group at C-3.

### Computational details

Ab initio calculations were carried out with the Gaussian 98 [33] for Linux, Gaussian 98 for Windows, and AIMAll [34] programs; visualization and geometry construction was done with the viewers Molden [35] and GaussView [36]. The geometries of the structures under study were optimized, relative energies, preferred structural conformations, dihedral angle scans, and frequency analysis were carried out at the HF/3-21G and B3LYP/6-311++G(d,p) levels of theory. The AIM analyses were carried out starting from the B3LYP/6-311++G(d,p) wavefunctions and geometries.

### Experimental

The imidazo[1,2-a]pyridine derivatives used in this investigation were prepared following the protocols described in the references shown in Table 1 and identified by comparison of their physical data with those reported in the literature. ^1^H and ^13^C NMR spectral data were recorded at 300 and 75 MHz respectively using a Bruker DPX 300 MHz NMR spectrometer. H-5 chemical shifts (δ) are given in parts per million downfield from TMS (δ = 0). The X-ray diffraction analysis was carried out in an Oxford Diffraction Xcalibur S diffractometer.

## Conclusion

Computational calculations predict correctly the conformational preferences in 3-π-EWG substituted imidazo[1,2-a]pyridines. When these π-EWG substituents meet the condition of being angular (aldehyde, acyl, nitroso), the thermodynamic equilibrium favors the conformer with the oxygen oriented towards H-5 of the imidazo[1,2-a]pyridine core. The existence of an electrostatic hydrogen bond between H-5 and the oxygen atom of the π-EWG is supported by the following facts: The geometries of the global minima already mentioned; the existence of critical points within the O···H inter-atomic space and the corresponding values of the electron density and the Laplacian of the electron density at these critical points; the magnitude of their interatomic distance is less than the sum of the van der Waals radii. With regards to the observed H-5 chemical shift caused by the angular EW groups at position 3, we concluded that this is an interplay of several factors which include the already mentioned electrostatic interaction along with a negative inductive EWG effect, anisotropy and long-distance mesomerism. The X-ray analysis performed on 3-nitroso-2-phenyl imidazo[1,2-a]pyridine, as well as other diffraction studies reported in the literature on analogous molecules, indicate that the solid-state geometry is closely related to the most stable conformational structures predicted by theoretical calculations.

## Competing interests

The authors declare that they do not have competing interests.

## Authors’ contributions

MVP synthesized most of the 3-EWG substituted imidazo[1,2-a]pyridines and under the guidance of HAJV carried out the dihedral angle calculations and the BCP analysis. HC, RJ and MECA carried out the ^1^H NMR spectra, discussed the ^1^H assignments and revised and gathered the references cited in the literature. TBH and HSZ built up and revised the manuscript and all together helped in writing the final version. MVP was a graduate student under the supervision of HSZ. All authors read and approved the final manuscript.

## Supplementary Material

Additional file 1 The X-ray diffraction data of compound 4. Click here for file

Additional file 2 ORTEP view of the asymmetric unit of compound 4. Click here for file
